# Preliminary experience of the isolate left subclavian artery *in-situ* fenestration during ‘zone 2’ thoracic endovascular aortic repair

**DOI:** 10.1093/ejcts/ezae332

**Published:** 2024-09-10

**Authors:** Gabriele Piffaretti, Andrea Gaggiano, Giovanni Pratesi, Valerio Tolva, Davide Pacini, Raffaele Pulli, Santi Trimarchi, Luca Bertoglio, Domenico Angiletta, AIDA (Ankura Italian Data Collection) Group, Gabriele Piffaretti, Andrea Gaggiano, Giovanni Pratesi, Valerio Tolva, Davide Pacini, Raffaele Pulli, Santi Trimarchi, Luca Bertoglio, Domenico Angiletta, Marco Franchin, Filippo Piacentino, Michelangelo Ferri, Simone Quaglino, Martina Bastianon, Davide Esposito, Nicola Monzio Compagnoni, Erika De Febis, Luca Di Marco, Giacomo Murana, Aaron Thomas Fargion, Sara Speziali, Chiara Lomazzi, Viviana Grassi, Irene Fulgheri, Stefano Bonardelli, Apollonia Verrengia, Sergio Zacà, Lucia De Santis, Gianfranco Veraldi, Luca Mezzetto, Diego Moniaci, Paolo Frigatti, Paola Scrivere, Alberto Dall’Antonia, Arnaldo Ippoliti, Stefano Fazzini, Yamume Tshomba, Giovanni Tinelli, Tiziano Porretta, Marco Tadiello

**Affiliations:** Vascular Surgery—Department of Medicine and Surgery, University of Insubria School of Medicine and ASST Settelaghi University Teaching Hospital, Varese, Italy; Vascular and Endovascular Surgery Unit, Mauriziano Umberto I Hospital, Turin, Italy; Vascular Surgery—Department of Surgical Sciences and Integrated Diagnostics, University of Genoa School of Medicine, Genoa, Italy; Vascular Surgery, Grande Ospedale Metropolitano Niguarda, Milan, Italy; Cardiac Surgery—Department of Medical and Surgical Sciences, University of Bologna School of Medicine, Bologna, Italy; Vascular Surgery, Department of Cardiothoracic and Vascular Surgery, University of Florence School of Medicine, Florence, Italy; Vascular Surgery, Department of Clinical Sciences and Community Health, University of Milan School of Medicine, Milan, Italy; Vascular Surgery, Department of Clinical and Experimental Sciences, University of Brescia School of Medicine, Brescia, Italy; Vascular Surgery, Department of Emergency and Organs Transplantation, University of Bari School of Medicine, Bari, Italy

**Keywords:** *In-situ* fenestration, Left subclavian artery fenestration, Adjustable needle, ‘zone 2’, TEVAR

## Abstract

**OBJECTIVES:**

To evaluate the results of isolated left subclavian artery *in-situ* fenestration (ISF) during ‘zone 2’ thoracic endovascular aortic repair (TEVAR) using a new adjustable needle puncturing device system.

**METHODS:**

It is a multicentre, retrospective, physician-initiated cohort study of patients treated from 28 July 2021 to 3 April 2024. Inclusion criteria were isolate left subclavian artery revascularization for elective or urgent/emergent ‘zone 2’ TEVAR. The primary outcome was technical success and freedom from ISF TEVAR-related reintervention or endoleak.

**RESULTS:**

We treated 50 patients: 28 (56.0%) atherosclerotic thoracic aneurysms, 12 (24.0%) type B aortic dissection and 10 (20.0%) penetrating aortic ulcers. Elective intervention was carried out in 46 (92.0%) cases. ISF was successful in all cases, with a procedural primary technical success in 47 (94.0%) cases. The median time of intervention was 184 min (interquartile range 135–220) with a median fenestration time of 20 min (interquartile range 13–35). Operative mortality did not occur. We observed 1 case of spinal cord ischaemia and 2 cases of bilateral posterior non-disabling stroke. Mortality at 30 days occurred in 1 (2.0%) patient (not aorta-related). The median follow-up was 4 months (interquartile range 1–12.25). Bridging stent graft patency was 100% with no ISF-related endoleak. ISF-related reintervention was never required

**CONCLUSIONS:**

ISF TEVAR using the Ankura™-II device with the self-centring adjustable needle system showed high technical success, promising stability and stable aortic-related outcomes. Owing to these results, it represents a safe and effective alternative for standard ‘zone 2’ TEVAR.

## Graphical abstract

**Figure:**
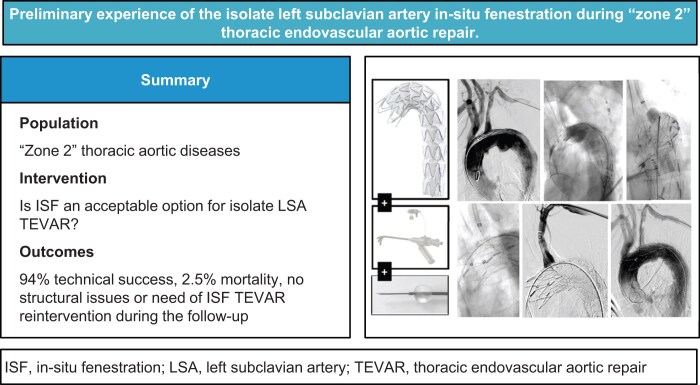

## INTRODUCTION

Meta-analyses have demonstrated that revascularization or preservation of the left subclavian artery (LSA) is associated with decreased risks of ischaemic and neurologic accidents [1–4]. Three cardiovascular societies identified left carotid-LSA bypass as the mainstay of treatment for LSA preservation during ‘zone 2’ thoracic endovascular aortic repair (TEVAR) [5–7]. Totally endovascular solutions have generated interest in the last decade; however, though versatile, parallel grafts techniques have shown meagre long-term effectiveness, customized fenestrated or scalloped or branched endograft (EG) require long manufacturing time, and physician-modified EG are technically demanding [8–11]. First proposed as bailout procedure in 1994, *in-situ* fenestration (ISF) of the LSA combines several attractive aspects: it creates an anatomical anterograde reconstruction and thus mimics open repair, it is based on simple technical steps being versatile for urgent/emergent scenarios [12, 13]. Among different ISF techniques, a self-centring adjustable needle-based puncture device was recently designed to improve the safety and success rate of ISF [14]. The aim of the present study was to evaluate the results of isolate LSA ISF during ‘zone 2’ TEVAR using this device in a real-life, recent cohort of thoracic aortic diseases.

## METHODS

### Ethical statement

Helsinki Declaration and its later amendments were respected. This type of procedure has been described, and in-depth specifically analysed with each patient during joint multidisciplinary discussions, and not before having described the possible technical alternatives. All these patients presented peculiar demographic and/or anatomical characteristics that were not adequately treatable with alternative techniques, thus resulting ISF the safest method to treat these lesions. The study was approved by the local Ethics Committee (Nr. 150/24) and registered as an observational study.

### Data availability

Data entry was managed by physicians involved into patient care, and merged into this single dataset by the corresponding author (G.Pi.). The data underlying this article will be shared on reasonable request to the corresponding author.

### Study cohort

This is a multicentre, financially unsupported physician-initiated, registry-based, observational cohort study [15]. The registry has now gathered data from 16 centres. Information collected included demographics, co-morbidities, morphologic characteristics of the aortic lesion, type of intervention and endograft as well as postoperative events (complications, death, aorta-related reintervention) during hospitalization and follow-up. The study period includes TEVAR performed between 28 July 2021 and 3 April 2024. The only 1 inclusion criteria was:

- isolate LSA revascularization with ISF during ‘zone 2’ TEVAR using the new adjustable needle puncturing system (FuThrough^TM^, Lifetech Scientific, Shenzhen, China) in combination with the Ankura^TM^-II (Lifetech Scientific; Shenzhen, China) thoracic endograft.

Exclusion criteria were the following ones:

- LSA preservation/revascularization for ‘zone 0-1’ TEVAR
- ISF with different techniques and/or devices
- missing preoperative computed tomography angiography
- missing clinical and/or procedural data.

### Aortic imaging assessment

Thoracic aortic disease was diagnosed in all cases by computed tomography angiography. Aortic sizing was determined by the aortic neck diameters, lengths and angles at the level of the supra-aortic trunks. Diameters were measured outer to the outer layer at the intended proximal landing ‘zone 2’. Lengths were calculated as inner/centreline/outer curvatures stretched length between the distal edge of the left common carotid artery and the proximal edge of the LSA or aortic neck. The distance between the LSA and the ipsilateral vertebral artery take-off was intended as stretched centreline length between the take-off of the LSA and the origin of the vertebral artery. Left subclavian artery angulation was evaluated in the best multiplanar projection of the arch. Aortic anatomy analysis was evaluated using a dedicated software (Intuition^TM^, Terarecon, Durham, NC, USA).

### Procedural details

**Figure 1:**
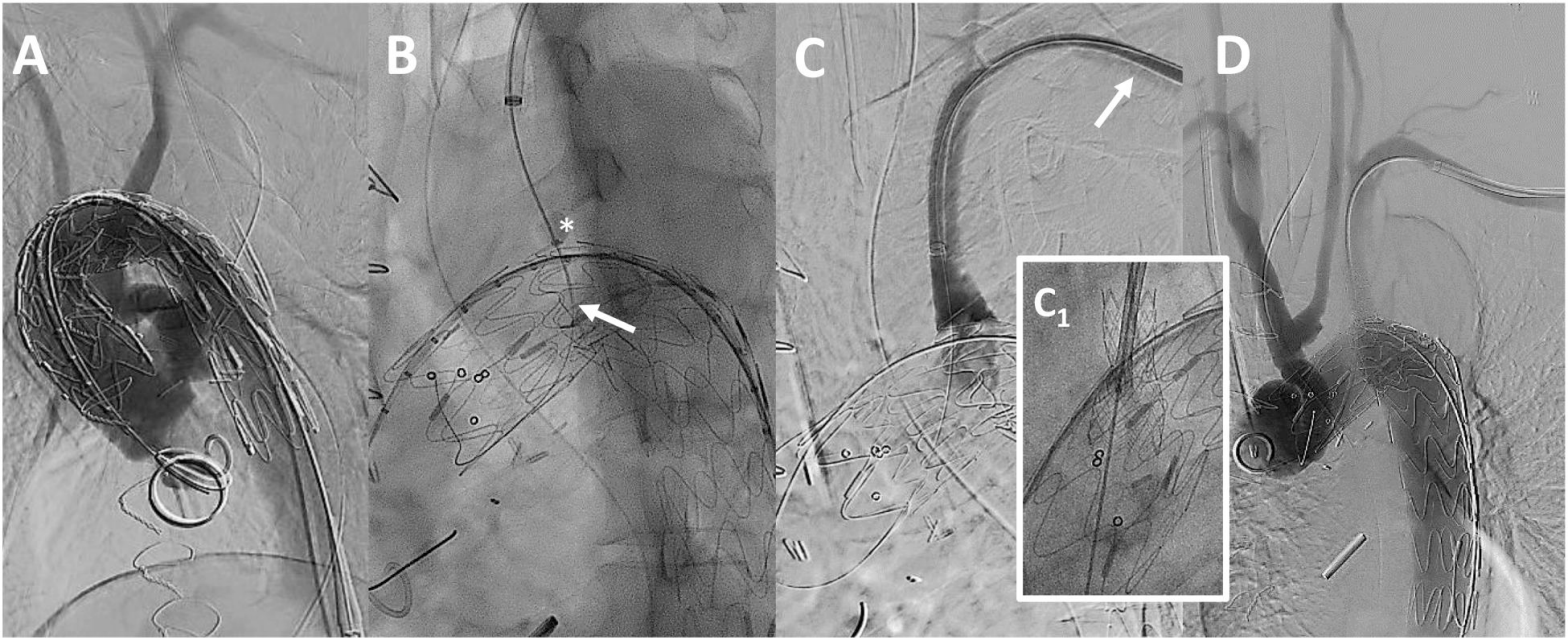
Intraoperative angiographic steps of ISF TEVAR for a descending thoracic post-dissection aneurysm (case #26). EG deployment (**A**). Needle-driven (white arrow) fenestration through a balloon-stabilized steerable sheath positioned in direct contact with the outer curvature of the EG (**B**, asterisk). Balloon dilatation of the ISF (**C**) and (n.2) bridging stent graft connection (C_1_) with anterograde protrusion to the ascending aorta due to persistent false lumen patency (white arrow) in the LSA. Final completion angiography (**D**). EG: endograft; ISF: *in-situ* fenestration; LSA: left subclavian artery; TEVAR: thoracic endovascular aortic repair.

**Figure 2:**
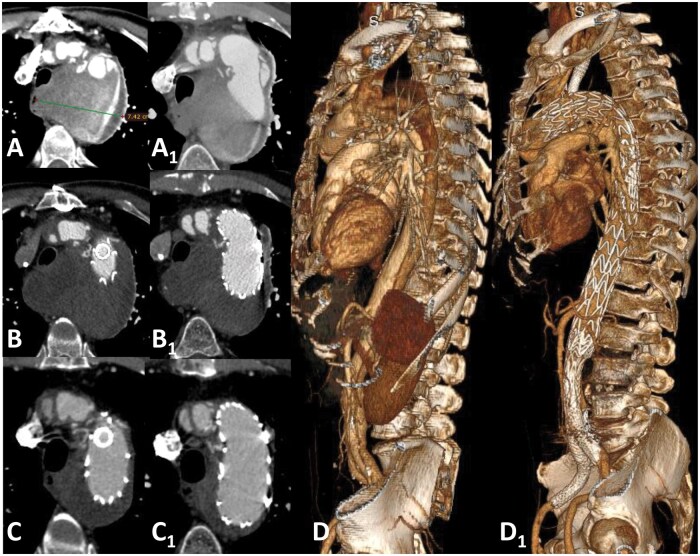
Preoperative CT-A (**A**–**C**) and 12 months follow-up CT-A (**A_1_**, **B_1_**, **C_1_**) of the descending thoracic post-dissection aneurysm (case #26) showing the complete shrinkage of the sac. Preoperative (**D**) and 12 months follow-up (**D_1_**) volume-rendering 3D CT-A. CT-A: computed tomography-angiography.

We shared a standardized technique: after deployment of the Ankura™-II thoracic endograft (Fig. 1A), a steerable sheath (Fustar^TM^ 5–10 Fr/550–900 mm in length—Lifetech Scientific, Shenzhen, China) is inserted through a left axillary artery access (either percutaneously or through a surgical cutdown) and positioned as perpendicular as possible to the greater curvature of the aortic arch (Fig. 1B). Then, the balloon at the tip of the puncture system is inflated possibly in direct contact with the outer curvature of the endograft and adjusted in length to make the fenestration safe and stable. After the fenestration is created, it is gently and progressively dilated using 4–8 mm diameter non-compliant balloons (Fig. 1C). Finally, ISF is connected with a balloon-expandable bridging stent graft (BeGraft/BeGraft^®^ plus, Bentley, Hechingen, Germany or VBX^®^, W.L. Gore & Associates, Flagstaff, AZ, USA) with a 10% oversizing, possibly deployed antegrade up to 5 mm inside the fenestration (Fig. 1C_1_ and D). During the follow-up, triple-phase computed tomography angiography follow-up was performed at 1 month and 12 months, and on an annual basis thereafter (Fig. 2).

### Definitions and primary outcomes

Morphological characteristics and outcomes were defined based on the European Association for Cardio-Thoracic Surgery (EACTS) best practice guidelines for reporting treatment results in the thoracic aorta and/or the Society for Vascular Surgery (SVS) *ad hoc* committee on TEVAR reporting standards [16, 17]. Aortic arch configuration was classified according to Ou *et al.* [18] and also using the MALAN stratification [19]. Specifically for the purposes of this study, fenestration time was defined as the interval time between upper limb access vessel puncture to final bridging stent graft deployment. The follow-up index described follow-up completeness at a given study end date as ratio between the investigated and the potential follow-up period [20]. The classification of complication severity adhered to the SVS definitions grading system. Specifically for this study, we concentrated on neurologic complications (e.g., cerebrovascular, spinal cord ischaemia), and device-related issues (endoleaks, migration, bridging stent graft instability) [21]. Specifically for this study, primary outcomes were technical success, survival and freedom from ISF TEVAR-related reintervention. Secondary outcomes were freedom from ISF-related endoleak and bridging stent graft instability [21].

### Outcomes and statistical analysis

Statistical analysis was performed with SPSS, release 27.0 for Windows (IBM SPSS Inc.; Chicago, IL, USA) [22]. Continuous variables were tested for normal distribution by the Shapiro–Wilk test and compared between groups with unpaired Student’s *T*-test for normally distributed values; otherwise, the Mann–Whitney *U*-test was used. Continuous variables that presented normally distributed were reported as mean ± standard deviation with range, otherwise with medians and interquartile range (IQR) (25–75th percentile interval) were applied. Categorical variables were reported as counts and percentages. All reported *P* values were two-sided; *P* value <0.05 was considered significant.

## RESULTS

### Study population

**Figure 3:**
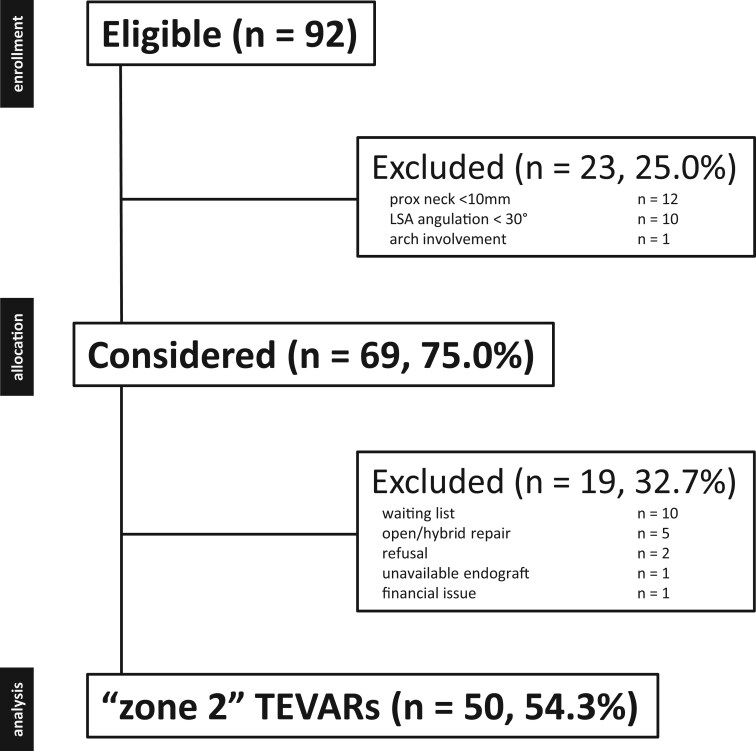
Consort diagram of all thoracic aortic diseases screened for ISF TEVAR (2021–2024, *n* = 81). ISF: *in-situ* fenestration; TEVAR: thoracic endovascular aortic repair.

We treated 50 (54.3%) patients of the whole aortic cohort evaluated for TEVAR with isolate ‘zone 2’ ISF (Fig. 3): 40 (80%) males and 10 (20%) females. The median of age was 74 years (IQR 68–79.25). Demographic data, comorbidities and risk factors are summarized in Table 1. We treated 28 (56.0%) atherosclerotic thoracic aneurysms, 12 (24.0%) type B aortic dissection and 10 (20.0%) penetrating aortic ulcers. The mean aortic lesion diameter was 56 ± 11 mm (range 39–76).

**Table 1:** Demographic data, coexisting comorbidities and risk factors of the entire cohort

	ISF for ‘zone 2’ TEVAR
	(*n* = 50)
Demographics	
Gender, M:F (ratio)	40:10
Age, median (IQR)	74 (68–79.25
Age ≥ 80 (years), *n* (%)	13 (26)
Comorbidities, *n* (%)	
Hypertension	41 (82.0)
Dyslipidaemia	28 (56.0)
Chronic obstructive pulmonary disease	24 (48.0)
Coronary artery disease	15 (30.0)
Chronic kidney disease	15 (30.0)
Diabetes	14 (28.0)
Synchronous AAA	9 (18.0)
Atrial fibrillation	9 (18.0)
Obesity	9 (18.0)
Risk factors, *n* (%)	
Urgent/emergent	4 (8.0)
Previous cardiac surgery	10 (20.0)
Previous aortic surgery	12 (24.0)
AbF bypass	4
AAA graft replacement	2
EVAR	4
TEVAR	2
FEVAR	1
SVS score [33], median (IQR)	6 (5–13.7)
EuroSCORE-I [17], median (IQR)	11.8 (5.4–18.3)
EuroSCORE-II [17], median (IQR)	3 (1.6–6.3)

AAA: abdominal aortic aneurysm; AbF: aorto-bifemoral; EVAR: endovascular abdominal aortic repair; F: female; FEVAR: fenestrated endovascular aortic repair; IQR: interquartile range; ISF: *in-situ* fenestration; M: male; SVS: Society for Vascular Surgery; TEVAR: thoracic endovascular aortic repair.

### Aortic anatomical features

Aortic arch morphology was normal in 38 (76.0%), crenel in 7 (14.0%) and gothic in 5 (10.0%). A type III aortic arch configuration was present in 19 (38.0%) of the patients. An anatomical variant of the supra-aortic trunks was observed in 8 (16.0%) cases (common innominate-left carotid *n* = 7, isolate left vertebral artery *n* = 1). Vertebral artery dominant side was left in 30 (60.0%) cases, and right in 20 (40.0%). Morphologic features at the proximal and distal landing zone are reported in Table 2.

**Table 2:** Morphologic features of the aortic lesions in the entire cohort

	ISF for ‘zone 2’ TEVAR
	(*n* = 50)
Morphologic features	
Proximal fenestration neck, median (IQR)	11.35 (9.4–15)
Proximal aortic neck length, mean ± SD (range)	24.3 ± 6.8 (12–38)
Proximal aortic neck diameter, mean ± SD (range)	31.5 ± 3.6 (21–38)
Distal aortic neck diameter, mean ± SD (range)	30.1 ± 6 (14–50)
Proximal LSA diameter, median (IQR)	12 (11–13.9)
Distal LSA diameter, median (IQR)	9.9 (834–11.5)
LSA take-off angle, median (IQR)	63 (50–91.5)
LSA-LVA length, median (IQR)	41.5 (36–50)

IQR: interquartile range; ISF: *in-situ* fenestration; LSA: left subclavian artery; LVA: left vertebral artery; SD: standard deviation; TEVAR: thoracic endovascular aortic repair.

### Procedural details

Elective intervention was performed in 46 (92.0%): we treated 4 (8.0%) cases in urgent setting due to an acute aortic syndrome caused by an acute type B aortic dissection complicated by refractory pain and/or rapid enlargement. Endograft was deployed under permissive hypotension in 46 (92.0%) cases (drug-induced *n* = 34; rapid pacing *n* = 12). ISF was successfully performed in all the cases, and the primary technical success was obtained in 47 (94.0%) cases: in 3 (6.0%) cases, a bailout stenting was needed for an unintentional partial coverage of the left common carotid artery. Indeed, in all these latter cases, this was not determined by EG misplacement, but correlated with the need of fully exploiting the proximal landing zone in steep aortic arches. Additional intraoperative or in-hospital procedures are reported in Table 3. Median time of intervention, including additional procedures, was 184 min (IQR 135–220) with a median fenestration time of 20 min (IQR 14–35). The median aortic coverage was 200 cm (IQR 160–310), with the distal landing zone below T_6_ in 35 (70.0%) cases, and between T_11_ and L_2_ in 13 (26.0%).

**Table 3:** Technical details and adjunctive procedures

	ISF for ‘zone 2’ TEVAR
	(*n* = 50)
Device measurements	
Endograft proximal LZ diameter, mean ± SD (range)	37 ± 4 (30–44)
Tapered, *n* (%)	30 (76.9)
Bridging stent graft diameter, median (IQR)	10 (10–12)
Bridging stent graft length, median (IQR)	39 (37–57)
BeGraft^®^ (Bentley)	48 (96.0)
VBX^®^ (W.L. Gore)	2 (4.0)
Additional procedure	
LCCA bailout stenting	3
BEVAR	3
distal TEVAR	2
FEVAR	1
Iliac stenting	2
Axillary stent	2
LVA thromboaspiration	1
SFA stent graft	1
SMA/RRA stent	2
Femoral endarterectomy	2
Infrarenal aortic ‘double-barrel’ relining	1
Infrarenal aortic thromboaspiration	1

BEVAR: branched endovascular abdominal aortic repair; FEVAR: fenestrated endovascular aortic repair; IQR: interquartile range; ISF: *in-situ* fenestration; LCCA: left common carotid artery; LVA: left vertebral artery; LZ: landing zone; SD: standard deviation; SFA: superficial femoral artery; SMA/RRA: superior mesenteric artery/right renal artery; TEVAR: thoracic endovascular aortic repair.

### Early outcomes (<30 days)

Operative mortality did not occur. Intraoperative endoleaks were not observed: specifically, initial type 3 endoleak was never observed. Forty-seven (94.0%) patients were transferred to the intensive care unit for a median of 17 h (IQR 10.2–38). We observed a postoperative major complication in 9 (18.0%) patients (Table 4): neurologic complications occurred in 3 (6.0%), 1 case of spinal cord ischaemia in an aortic coverage below T_12_ and 2 bilateral posterior non-disabling strokes. Regarding strokes, they occurred in patients with severely atheromatous aortic arch. The median of hospitalization was 6 days (IQR 5–15.75), being significantly higher in complicated patients (days, 16 vs 5, *P* = 0.050). Mortality at 30 days occurred in 1 (2.0%) patient (acute on chronic respiratory insufficiency *n* = 1).

**Table 4:** Postoperative complications

Type of complication	Cases (*n*)	SVS [33] (severity grade)	Treatment
Neurologic			
Posterior stroke^a^	2	3	Rehab
Spinal cord ischaemia	1	3	Rehab
Cardio/respiratory			
Pneumonia	2	2	Antibiotics
Atrial fibrillation	1	2	Amiodarone
Pleural effusion	1	1	Conservative
Local			
Brachial/axillary dissection	2	2	Stenting

SVS: Society for Vascular Surgery.

a Bilateral and non-disabling.

### Follow-up outcomes

Of the 49 patients discharged alive, none was lost at a median clinical and radiologic follow-up of 4 months (IQR 1–12.25). Specifically, 32 (64.0%) had a follow-up within 6 months, 14 (28.0%) between 6 and 12 months and 13 (26.0%) beyond 12 months. The median of follow-up index was 0.65 (IQR 0.28–1.0). During the course of the follow-up, no aorta-related mortality was observed; only 1 (2.0%) patient died at 5 months for TEVAR-unrelated cause (urinary sepsis). At the last follow-up available, bridging stent graft patency was 100% with no ISF-related endoleak. No breakage, fracture or migration of the bridging stent graft was observed. We did not observe TEVAR failure due to device integrity issues. ISF-related reintervention was never required; aorta-related reintervention occurred in 2 (4.1%) patients when a dissection-related re-entry tear was sealed-off with an additional distal extender endograft at 4 and 9 months, respectively.

## DISCUSSION

The main findings of this preliminary multicentre experience are that ISF for ‘zone 2’ TEVAR is satisfactorily feasible, safe and a durable repair at early follow-up.

While surgical bypass remains the mainstay of treatment for ‘zone 2’ TEVAR, modern clinical surgery is committed towards the development of minimally invasive techniques while maintaining effectiveness and durability [7]. Two systematic reviews recently ascertained the high technical success rates and satisfactory results in the short term with different ISF techniques [12, 13, 23]. Our experience is one of the few to analyse the clinical results of ISF TEVAR with self-centring adjustable needle on a homogeneous cohort based on a combination of specific devices, whose results are in line with an overview of the available literature showing a satisfactory high technical success (94–98%) with acceptably low mortality as well as stroke rate (<3%) in comparison with those reported for carotid-subclavian bypass or parallel grafts [24–28]. Despite the multicentre nature of our registry-based study, outcomes were homogeneously safe and effective across the different participating centres, since they were based on careful morphologic case selection, meticulous respect of the instructions for use and the homogeneous combination of dedicated devices.

Structural integrity and outcomes durability are cornerstone parameters to evaluate new technologies or technical alternatives. Among total endovascular solutions for ‘zone 2’ TEVAR, branched and fenestrated endografts have the better possibility of being tailored for each patient’s anatomy. Nonetheless, they are expensive and technically demanding with no immaculate results in terms of neurologic sequelae (2.6–3.4%) and reintervention either (0–2.6%) [8–11]. In our ongoing experience, early results are sincerely promising with no issue at all regarding bridging stent graft integrity, patency, migration and endograft fabric damage. These clinical results resemble the favourable findings of different bench studies; Jayet *et al.* [29] demonstrated that laser fenestration expose fabric to a higher risk of mechanical resistance loss and leakage at the stent–fenestration interface when fabric debris and toxic particles due to burning of the material are generated. In a comparative analysis, Li *et al.* [30] demonstrated that the combination of needle-driven ISF with the Ankura^TM^-II endograft produces the highest score and best shape of fenestration with the best stable margins of holes without fabric tears, also over a simulated 10-year period [31]. Lastly, despite potential differences that may exist across countries, at least in our country, ISF is currently cheaper than branched and fenestrated endografts.

In previous clinical experiences, one of the most important anatomic constraints for using ISF was the aortic arch configuration, a finding that is a common issue for other types of complex TEVAR technologies to be used in this area [32]. Shu *et al.* [25] reported that both the acute take-off angle of the LSA and the severe tortuosity rendered to be constraining factors for ISF. However, while dissection was the predominantly treated disease in the clinical experiences reported so far with these ISF techniques, atherosclerotic aneurysm was the main indication in our series [24–28]. This difference may be critical for the ISF feasibility. On the one hand, type B aortic dissection has been frequently associated with the gothic conformation of the aortic arch, in aneurysmal disease, the angulations of the supra-aortic trunks’ origin are often challenging, a factor that has been shown to be critical for ISF [19]. The adequately high technical success rate obtained in the various experiences reported in the literature, as occurred in our preliminary one, testifies to the validity of ISF as an alternative technique for the preservation of the LSA, also emphasizing the fact that a type III aortic arch is not an absolute contraindication *per se* performing ISF.

Last but not least, regulatory and legal consequences should be kept in mind when treating patients; ISF TEVAR is not exempt of complications or structural concerns along the follow-up time, similar to other totally endovascular solutions [11, 34]. Regulatory does not prohibit the use of modified medical devices, unless justified by healthcare professionals based on clinical evidence and patient needs when suitable on-label alternatives are not available, and only when they believe it is in the best interest of the patient [35]. As far as these two aspects are concerned, not only ISF TEVAR has plenty of literature that demonstrates satisfactory results in line with other accepted alternative techniques, but in our experience, these cases were discussed in multidisciplinary evaluations with each patient who accepted the technical proposal based on the fact that other technical alternatives were not affordable either from a technological or technical or anatomical point of view [2, 3, 13]. In addition, EGs modified at bench by physicians are highly complex from a technical standpoint, and lacks codified procedural guidelines, while ISF TEVAR is based on a few and coded steps that, at least in our experience, were shared mandatorily by all centres and are technically more similar to those used in the more standardized fenestrated or branched procedure [36].

### Limitations

This study has several limitations. First, it is retrospective in nature. Since each institutional database relies solely on the accuracy and completeness of data collection, it is possible that in such context, investigators might have not identified all patients and all variables. However, missing data were not defaulted to negative, and denominators reflect only reported cases: from the total of 50 patients, 3200 data were collected across 64 variables, with an overall missing data rate of 0.8%. Second, it has sampling bias: alternative fenestration techniques were not investigated for comparison. Third, while we attempted to correct for potential confounders using multivariate analyses, the small number of patients and events makes the results not generalizable.

## CONCLUSION

In our experience, ISF TEVAR using the Ankura™-II device in addition with the self-centring adjustable needle system showed very high technical success, with promising device stability and stable major aortic-related outcomes in the early-to-midterm follow-up. Owing to these results, it represents a safe and effective alternative for ‘zone 2’ TEVAR.

## Data Availability

Data are available on reasonable request.

## ABBREVIATIONS

EACTS: European Association for Cardio-Thoracic Surgery
EG: Endograft
IQR: Interquartile range
ISF: *In-situ* fenestration
LSA: Left subclavian artery
SVS: Society for Vascular Surgery
TEVAR: Thoracic Endovascular Aortic Repair
